# Session 1: DNA repair

**DOI:** 10.1093/jrr/rrt206

**Published:** 2014-03

**Authors:** George Iliakis

**Affiliations:** Institute of Medical Radiation Biology, University of Duisburg-Essen Medical School, Hufelandstraße 55, 45122 Essen, Germany

## SESSION SUMMARY

The first plenary session of the meeting was dedicated to DNA repair. Investigators from six outstanding groups in the field were invited to present their latest findings. Speakers in the order of appearance included Dr Takashi Morita from Osaka City University in Japan, Dr Erica Werner from the group of Dr Paul Doetsch at Emory University in the USA, Dr Akinari Yokoya from the Japan Atomic Energy Agency, Dr Susan Bailey from Colorado State University in the USA, Dr Sandro Conrad from the group of Dr Marcus Lobrich at Darmstadt University of Technology in Germany, and Dr Aroumougame Asaithamby of the University of Texas Southwestern Medical Center in the USA. Overall the talks focused on aspects of cellular responses to DNA damage as they relate to space radiation biology for missions to the International Space Station (ISS), the moon or Mars, as well as to ion radiotherapy.

The presentation of Dr Morita focused on the detection of chromosome aberrations in γ-H2AX-proficient and -deficient mouse embryonic stem (ES) cells in the space environment of the Japanese experimental module ‘KIBO’ at the ISS [1]. It is planned that cells will be flown to KIBO and stored there for up to 3 years in a frozen state (at − 95°C) and will be periodically returned to Earth (overall five times), where analysis for chromosome aberration formation from space radiation will be carried out. Notably, radiation-exposed and control ES cells can also be microinjected into unirradiated embryos, and surviving embryos can be implanted into pseudo-pregnant mice to analyze for developmental defects induced by space radiation. The authors provided evidence that all related assays required for this set of experiments are already developed in their laboratories. The first experiments were therefore designed to validate the potential of the system. For this purpose, the investigators characterized the selected mouse ES cells using γ-rays or ^56^Fe-ions and measured chromosome aberrations by fluorescence *in situ* hybridization (FISH). The results obtained in these validating experiments demonstrated increased radiosensitivity compared with wildtype H2AX-deficient cells, and this was confirmed by analyzing certain forms of chromosomal aberrations. This validated system of ES cells can now be used for the quantitative estimation of the biological consequences of space radiation. The actual space radiation experiment started in March 2013 by flying wildtype and histone H2AX-deficient ES cells to ISS. This is the first experiment of its type designed to directly examine the effect of the space radiation environment at different endpoints. Although the ISS is not receiving the spectrum of particles expected in deep space, and cells in a frozen state sustain a different spectrum of lesions in their DNA than non-frozen cells, the results are awaited with great interest.

The presentation of Dr Erica Werner focused on the role of Reactive Oxygen Species (ROS) in the resolution of persistent genomic instability following exposure to radiation [2]. This work is based on the hypothesis that ROS generated as a consequence of a radiation exposure can amplify the initially induced radiation damage sustained by macromolecules. It is further considered that ROS can amplify downstream responses to DNA damage that determine DNA repair and cell death. ROS are even thought to amplify delayed radiation responses leading to tissue damage and/or tumorigenesis. Results were presented showing that in immortalized normal human bronchial epithelial cells (HBEC-3KT) exposed to X-rays or ^56^Fe-ions, increased ROS levels can persist in surviving cells for up to eight population doublings (2 weeks). It was observed that this increased ROS production overlapped temporally with the persistence of reporters for genomic instability, proliferation and senescence, and was associated with increased frequency of micronucleus formation and the presence of γH2AX-53BP1 foci. Although low-LET radiation at high doses and high-LET radiation induced a senescence-like phenotype dependent on ATM and p38 MAPK activity, ATM or p38 MAPK activation was not the cause of elevated ROS generation. Notably, inhibition of ATM or p38 MAPK further increased ROS levels and this resulted in a reduction in micronucleus formation, suggesting a form of adaptation. This interesting observation could be reproduced by exposure to exogenous hydrogen peroxide, which again caused a reduction micronucleus formation following irradiation. These intriguing results implicate ROS as an effector in the resolution of genomic instability and suggest interplays between ROS levels and the DNA repair machinery that require further investigations.

Clustered DNA damage generated by low-LET radiation, and to an even greater extent by high-LET radiation, is considered the main cause of the adverse effects of ionizing radiation (IR), including cell killing and carcinogenesis. Such clusters can contain several forms of lesions in close proximity including single-strand breaks (SSBs), apurinic or apyrimidinic (AP) sites, and purine or pyrimidine base lesions. In his presentation, Dr Yokoya provided a theoretical extension [3] of recently completed experimental work in which the yields of AP lesions and clusters, base lesions, strand breaks and base lesion clusters was studied using an *in vitro* plasmid assay after high- and low-LET radiation under conditions of varying radical scavenging capacity [4]. A theoretical model was developed to calculate the production of AP sites, strand breaks and base lesions following carbon ion exposure using a Monte Carlo track structure simulation code (TRACION) for ion tracks [5]. The calculations use as a model a 150-bp-long linear DNA molecule, and the scavenging capacities of the samples are varied to approximate the experimental conditions employed. The authors make the interesting assumption that AP sites form from the same initial OH radical adduct that also evolves to form SSBs and base lesions, and use newly determined branching ratios for their calculations: induction of AP sites to SSBs or base lesions of 17:33 or 2:48, respectively. Notably, while the calculated yields of AP sites and SSBs were consistent with available experimental data, the calculated yields for AP clusters and double-strand breaks (DSBs) under low scavenging capacities were one-fifth of those experimentally measured. Dr Yokoya and his collaborators suggested that in order to bridge this discrepancy between theoretical and experimental yields, new models of DNA damage-clustering need to be developed that consider dissociative low-energy electron attachment, or hole-migration. The development and the testing of theoretical models with such capabilities are likely to advance our understanding of the mechanisms underlying the induction of clustered DNA damage.

Dr Bailey's talk focused on the role of telomeric proteins in the cellular responses to DNA damage induced by high- and low-LET IR, further developing the argument that telomere dysfunction contributes to genomic instability and cancer risk [6]. For this set of studies, the expression or activity of key telomeric proteins was reduced using small interfering RNA (siRNA), or small molecule inhibitors, and mutational or cytogenetic approaches were used to analyze phenotypic and genotypic consequences. Results were presented showing that deficiencies in TRF1, TRF2 and particularly POT1 significantly elevated spontaneous and IR-induced mutation frequencies (MFs) in a LET-dependent manner. TRF2 and POT1 deficiencies also resulted in telomere uncapping, evidenced by elevated telomere fusion. Depletion of the telomere-associated tankyrase1 elevated spontaneous and IR-induced MFs, as well as telomere sister chromatid exchange (T-SCE) frequencies in telomerase-deficient backgrounds. Notably, tankyrase1 PARP activity protected DNA-PKcs from proteasome-mediated degradation, thus identifying a novel aspect of DNA-PKcs regulation [7]. Experiments were also presented probing the non-tumorigenic human mammary epithelial MCF-10A and WTK1 B-lymphoblastoid cell lines for mechanistic links between stem cell-associated Wnt/β-catenin signaling and regulation of telomerase activity following IR exposure. A preliminary evaluation at various times after γ-ray exposure demonstrated stabilization of Wnt/β–catenin protein levels at 0–24 h, elevation of telomerase activity at 1–2 days, and enrichment of stem cell compartments at 5 days. The potential oncogenicity that may arise from such IR-induced cellular reprogramming of normal cells, and the role of DNA repair, telomere deficiencies, telomere length modulation and LET effects are under scrutiny. These intriguing studies continue to support the view that telomeres aren't just for telomeres but represent central determinants of DNA repair, aging and cancer risk. This line of investigation is exciting and likely to generate important information that will advance our understanding of radiation-induced cancer risk.

Dr Conrad addressed in his talk the influence of end-resection by CtIP on Artemis-dependent DSB repair in G1 and G2 phase after high- and low-LET radiation [8]. The premise of the work presented was that during the G1 and G2 phases of the cell cycle, a small fraction of DSBs that are located in heterochromatic regions are repaired with slow kinetics [9], and that this slow repair activity represents the function of homologous recombination repair (HRR) in G2 and of NHEJ in G1 [10]. Notably, deficiency of the Artemis endonuclease compromises the slow component of DSB repair by an unknown mechanism, both in G1 and in G2 phases. In the work presented the investigators tested the hypothesis that Artemis processes secondary or cruciform structures arising during DSB resection, specifically in heterochromatin. To test this prediction they depleted both CtIP (a factor necessary for the initial DSB end resection) and HRR in an Artemis-deficient background, and irradiated the cells with either X-rays or carbon-ions. Surprisingly, CtIP depletion rescued the Artemis repair defect both in G1 as well as in G2 phase following exposure to X-rays, suggesting that in the absence of CtIP DSB repair can proceed independently of Artemis. In contrast, after exposure to high-LET radiation, depletion of CtIP in an Artemis-deficient background causes a pronounced repair defect both in G1 as well as in G2 phase. These observations led the authors to suggest that following exposure to high-LET radiation, not only heterochromatic but also euchromatic DSBs undergo resection in G1 or G2 phase, in this way enhancing the requirement for Artemis activity. These intriguing observations suggest LET-dependent shifts in end resection between euchromatic and heterochromatic regions that alter the balance between HRR and the pathways of end-joining and thus also the requirements for Artemis activity. Further in-depth investigations are needed to address this exciting possibility and are ongoing in the laboratories of the investigators.

Dr Asaithamby's talk [11] focused on the function of the Fanconi anemia (FA) pathway in genome stability following exposure to high-LET radiation that induces clustered DNA lesions that are difficult to repair. The premise of the presentation was that although both NHEJ and HRR are involved in the repair of simple DSBs, it remains unknown whether multiple DNA repair pathways collaborate during repair of DSBs associated with clustered lesions. To address this very important point the investigators examined the possibility that the FA pathway, through its associated multiprotein complex, contributes to the decision to channel DSBs to HRR in favor of the competing error-prone NHEJ pathway. Results of experiments carried out with FA-deficient human cells were presented, suggesting that the FA pathway plays a key role in cellular responses to iron particle radiation. A novel live cell imaging technology showed that FA pathway factors are recruited to the sites of clustered DSBs, but only in a specific phase of the cell cycle. In addition, FA pathway–deficient cells become hypersensitive to iron radiation and exhibit higher numbers of unrepaired clustered DSBs. Notably, a DNA combing approach shows that FA pathway defects compromise the recovery of stalled DNA replication forks in iron-irradiated cells and alter the dynamics of RPA/RAD51 proteins at the sites of clustered DNA lesions. In line with these observations FA-deficient cells show higher, clustered lesion-proportional levels of chromosomal aberrations compared with wildtype cells that correlate with cellular transformation in a soft-agar transformation assay. The authors conclude that FA factors coordinate the processing of clustered DNA lesions to maintain genome stability in human cells. These are new and exciting observations that require further in-depth investigations.

## CONFLICT OF INTEREST

The author declares that there is no conflict of interest.

## FUNDING

This work supported by a grant from the “Bundesministerium für Wirtschaft und Technologie” (BMWi: ESA-AO-08-IBER, 50WB1229).
